# The Institutional Library

**Published:** 1920-03-06

**Authors:** 


### THE INSTITUTIONAL LIBRARY.

**Oto-Rhino Laryngology for the Student and Practitioner.** By Dr. Georges Laurens. Authorised English translation of the second revised French edition by H. Clayton Fox, F.R.C.S. (Ireland), with a foreword contributed by J. Dundas Grant, M.A., M.D., F.R.C.S. With 592 illustrations. (Bristol: John Wright and Sons, 1919. Pp. 10+339. Price 17s. 6d. net.)

This little book is intended as a guide to practitioners in the examination of the ear, nose, and throat, and in the treatment of the commoner disorders which come within the scope of the general practitioner. The book in general, but especially the sections on examination, is written with the most meticulous attention to detail, and would not be needed if all students attended the out-patient clinics of their throat and ear department; but, as things are, should prove useful to many doctors in practice who regret too late that they have not made use of their opportunities in this direction during their student days. The work is profusely illustrated with clever drawings and diagrams, and the writing is stimulating, concise, and to the point. It has, however, as is probably inevitable, the defects of its qualities; it is intensely dogmatic, and therefore often one-sided; it is decidedly uneven, devoting too much space to such subjects as gymnastic exercises, balneological treatment, and the detection of malingering, and too little to others such as the testing of hearing and the treatment of simple laryngitis, nasal obstruction, and other common affections which make up the bulk of practice in this speciality. In many cases it does not reflect the best British practice, and the doctor in this country will not find it everywhere a safe guide, notably where the practitioner is advised never to employ the guillotine for removal of tonsils, but to have recourse to "morcellement." The work of translation has been very well done, and there is an unusually full index.

### Some Public Health Publications.

The Welfare of the School Child. By Joseph Cates, M.D. English Public Health Series. Edited by Sir Malcolm Morris. Pp. 154. (London: Cassell and Co. 1919.)

Transactions of the Incorporated Sanitary Association of Scotland, 1918 (Bath Street, Glasgow).

Annual Report on School Hygiene for Warrington. By G. W. Joseph, M.D.

The simpler aspects of sanitary science have always appealed to English public opinion, and have consequently been paid attention to here because of their practical bearing. Such of these as concern looking after children of the public elementary schools are set out well enough by Dr. Cates in his manual. The layman or the health visitor will find it useful. It takes a properly progressive view of the requirements of working-class children. Meals, presumably free, at class-room canteens are desiderated, as also playing fields. We miss recognition of the fact, known to every general practitioner, that school attendance itself, under present conditions, is a great strain on the juvenile constitution. The rapidity with which a delicate child improves when excused school, and the frequency with which it relapses on return thither, prove this up to the hilt. The author is ill-advised in recommending the hospital system of junior resident and visiting honorary orthopædist at surgical tuberculosis institutions, since the treatment of that affection demands daily—nay, hourly—vigilance. Moreover, the results (as observed personally) in an institution of the kind recommended compare badly with those obtained at a place where the resident is in full charge. This little work covers the whole ground of school hygiene, but not intensively. The first important item in the transactions of the Scotch Sanitary Association is on a Ministry of Health for Scotland; and it is no surprise to find that the meeting voted overwhelmingly for the Secretary for Scotland to be nominated as Minister of Health for that country, with a Parliamentary Secretary in subordinate charge. It may be allowed that as regards Scotland, decentralisation in sanitary administration may be allowed to go thus far and no farther. Other features of this brochure, which is usefully bound in cardboard, and thus keeps its shape on a shelf, are discussions on pre-natal care and a valuable inquiry by Dr. Adam, of Sterling, into employment of children out of school hours. The spirit of the members seems to suffer a little from "war-hate." To judge by frequent expressions of opinion, Germany was the most backward country in the world in sanitation, with England a good second; and so these *comptes rendues* are food in the same way that mangoes are, which smack a little of turpentine, or rum, which (we are told) tastes of furniture polish. Dr. Joseph's report chronicles the usual activities of the up-to-date school medical officer. War conditions are reflected in an increase of children with verminous heads. An excellent feature is the separate report by the dental surgeon, whose work is, of course, of the greatest importance. Might it not be advisable, we ask him, to prescribe, when apples are cheaper, a small slice for each child, to be eaten *after* each meal, especially the last one of the day?

**Gynæcology for Nurses and Gynæcological Nursing** By Comyns Berkeley, M.A., M.D. (London: The Scientific Press. Price 6s. Third Edition, entirely rewritten, with illustrations.)

Few books of this type deal so understandingly with the pre-preparation, post-operative complication and the responsibilities relating to the nursing aspects of gynæcological surgery, together with advice both practical and helpful. The book gives a full synopsis of gynæcology, and has many descriptive illustrations also showing the arrangements of instruments required in operations of this nature. The writings of this author are too well known to need emphasising, and the present edition develops the clinical duties in detail that will be appreciated by every nurse who studies this branch of nursing.

**Psychologies.** By Ronald Ross. (London: John Murray. Price 2s. 6d. net.)

The essays in verse of a man of science must always command consideration, for that there is supposed to be an opposition between these two activities of the mind is seen in the extreme case of Charles Darwin, who declared that his scientific studies had led him to "nauseate" Shakespeare. Clearly this has not been the case with Sir Ronald Ross, who has received a respectful, and indeed an enthusiastic, hearing from modern poets. The five pieces in his latest volume are dramatic in form, and mainly written in blank verse, which reproduces several Elizabethanisms. Though murder and death play a large part in them, they are presumably meant to be character studies; but it is doubtful if they add

**The Institutional Library**—(*continued*).

anything to the melodramatic psychology from which Shakespeare set the drama free. As has been well said, he put our selves upon the stage, but not our situations. It would seem, then, that a true development of blank verse should revert neither to the situations of Elizabethan drama nor to the types of character peculiar to it, which Shakespeare did his best to humanise. Blank verse in his hands and those of Milton, Wordsworth, W. B. Yeats, and Thomas Hardy has produced such different effects that this demand does not appear impossible. Sir Ronald is most successful when he is most lyrical, and for this reason we like his verse best in his last two pieces. In "The Boy's Dream" he treats the ever-enchanting theme of Titania and Oberon gracefully, and that this seems to be better suited to his temperament than the more stormy effects of blank verse can be judged from the best line of the three preceding pieces :

"The deep endellèd woods are rich with flowers."

This at least is our verdict on his poetic "psychology."

**A History of the Scottish Women's Hospitals.** Edited by Eva Shaw McLaren. (Hodder and Stoughton. 7s. 6d. net.)

Dr. McLaren's book marks one of the highest collective achievements in the history of woman's work. It should be on the shelves of every woman's college, every training-school for nurses, for it demonstrates the success with which women doctors and nurses, assisted by lay women, can organise harmoniously and carry to a brilliant conclusion enterprises which tax energies, endurance, and skill to the utmost limit. The story of the Scottish Women's Hospital takes the reader first to Edinburgh, where the untiring zeal and determination of Dr. Elsie Inglis brought them into being in the teeth of official discouragement. Then the scene shifts to France, and we witness the rapid and efficient manner with which the Scottish women threw themselves into the first task set them—that of helping to combat enteric fever at Calais. Several intensely interesting chapters are devoted to the development of their fine hospital in the old Abbey at Royaumont. Then in Part III. the scene shifts to Serbia. The work of mercy carried out by the Scottish women in that country is part of the history of our times. Dr. McLaren succeeds in presenting events in clear sequence, with reticence and restraint, but she shirks none of the painful problems of the campaign. Her chapter on Elsie Inglis is one of the best appreciations of this noble woman we have seen. After the tragic retreat we are taken to Corsica, where good comfort was brought to the wounded refugees; then to Cetrovo, and on again with the transport columns till the S.W.H. made their entry with the triumphant army into Belgrade. The book is well documented, and contains a valuable appendix with medical and surgical reports of the work carried through. The illustrations are numerous and well produced, the print large and clear. A tabulated list of all who took a share in the work would have added to the value of this sincerely written record. The roll of honour includes the names of ten sisters and nurses.

**Arms and the Doctor.** By Henry Gervis. (C. W. Daniel. 1920. Pp. 86. Price 2s. 6d. net.)

Dr. Gervis served at home in a Territorial General Hospital for the first few years of the war, and was transferred to a base hospital in the Etaples district in the momentous spring of 1918, where he served until some few months after the Armistice. In a series of all too brief papers he conveys some of the more salient impressions of his military experience. His observation is acute, and his generalisations are sound, as far as they go. What the medical world is still waiting for is a more detailed and more reasoned account, from the civilian professional standpoint, of medical work in France; and it is a pity that Dr. Gervis cannot give us this, for his style is cultured and his judgments mature. The only slip that we have been able to find is his description of Camiers as situated in a valley.

**Wheeler's Handbook of Medicine.** By William R. Jack, B.Sc., M.D., F.R.F.P.S.G., Physician to Glasgow Royal Infirmary. (E. and S. Livingstone, 17 Teviot Place, Edinburgh Price 12s. net.)

We have to welcome the sixth edition of this very valuable little handbook, as although a reprint of the fifth edition appeared in 1918, yet the influence of the past war on accepted medical rulings has been so profound that in a student's examination guide such as this, a certain amount of modernisation was a necessity. The alterations have now been made, and although the form of publication remains essentially the same, much valuable new material has been incorporated. Also we note that the volume concludes with a section devoted to the more notable of medical disorders brought into especial prominence during the war.

**Practical Hints on Minor Operations.** By G. P. Mills, F.R.C.S. (Cornish Bros. 1920. Pp. 111. Price 5s. net.)

The operations dealt with are all minor and all common; they are of the type which any properly instructed medical man who has been house surgeon to a general hospital ought to find well within his capacity. The hints are also essentially practical. At the same time, it is doubtful whether so cursory a survey as this of minor operative surgery is likely to be satisfactory to those who may require advice on these matters. The teaching seems to us sound but too fragmentary.

**Food: Its Composition and Preparation.** By Mary T. Dowd and Jean D. Jamieson. (New York: Wiley & Sons; London: Chapman & Hall, Ltd. Price 6s.)

This food manual is one of the Wiley technical series for vocational and industrial schools, and is a text-book for classes in household science. It is of a nature to be very useful to sisters called on to give lectures on domestic science, for the information is given in a form which can be readily transmuted into class instruction. The chapters on bread and on cereal foods are particularly good. The book is plentifully illustrated, and some of the diagrams are novel and of great interest to the student. On the other hand, pictures of such common objects as cinnamon berries and brussel sprouts seem a little out of place. The expression "laboratory practice" as applied to the making of muffins and such like delicacies grates on the English ear.

**Elementary Organic Chemistry.** Adapted for the Use of Pharmaceutical and Medical Students. By F. Pilkington Sargeant. (London: H. K. Lewis and Co., Ltd. Price 4s.)

This simple textbook of organic chemistry now appears in its second edition and for the concise manner in which it deals with a subject always difficult to the student in his early days it is to be strongly recommended.

